# Adaptation of the Child Coeliac Disease Food Attitudes and Behaviours Scale (Child CD-FAB) into Brazilian Portuguese: Translation and Evaluation of Reproducibility and Internal Consistency

**DOI:** 10.3390/nu17162704

**Published:** 2025-08-21

**Authors:** Marina de Cesaro Schwantes, Heather Maddison-Roberts, Eduardo Yoshio Nakano, Raquel Braz Assunção Botelho, Renata Puppin Zandonadi

**Affiliations:** 1 Department of Nutrition, Faculty of Health Sciences, Darcy Ribeiro University Campus, University of Brasília, Brasília 70910-900, Distrito Federal, Brazil; raquelbotelho@unb.br (R.B.A.B.); renatapz@unb.br (R.P.Z.); 2 South West London & St George’s Mental Health NHS Trust, London SW17 0YF, UK; 3 Department of Statistics, University of Brasília, Brasília 70910-900, Distrito Federal, Brazil; nakano@unb.br

**Keywords:** hypervigilance around food, questionnaire, gluten-free diet, eating attitudes, eating behaviours

## Abstract

**Background/Objectives**: This study aimed to translate the Child Coeliac Disease Food Attitudes and Behaviours Scale (Child CD-FAB) into Brazilian Portuguese and to evaluate its reproducibility and internal consistency. **Methods**: Three steps were carried out: (i) translation and cultural adaptation of the Child CD-FAB into Brazilian Portuguese; (ii) pre-test; and (iii) psychometric evaluation of the Child CD-FAB-BR. The Child CD-FAB was translated and back-translated, and then discussed with and approved by the author of the original instrument. The translated version was tested on five children and adolescents (aged 8 to 16 years) with coeliac disease to assess comprehension. Subsequently, psychometric evaluation used responses from 20 participants with the same characteristics, assessing reproducibility (test–retest reliability) via intraclass correlation coefficient (ICC) and internal consistency via Cronbach’s alpha. **Results**: The Child CD-FAB was successfully translated to Brazilian Portuguese (Child CD-FAB-BR), and it presented good internal consistency (α = 0.892) and an excellent intraclass correlation coefficient (ICC = 0.923). **Conclusions**: The Child CD-FAB-BR is the first instrument to evaluate food attitudes and behaviours in Brazilian children and adolescents with CD, showing good psychometric properties. This instrument will support future research and clinical practice by guiding behaviour-based strategies to enhance nutritional education and public policies for this group.

## 1. Introduction

Initially considered a rare condition, coeliac disease (CD) has now been recognized as a global public health problem, with estimates of worldwide prevalence that have increased considerably over the last few decades, reaching approximately 1% of the general population, with variation according to sex, age, and geographic location. In the paediatric population, despite the sub-diagnosis, a systematic review showed that biopsy-confirmed prevalence is approximately twice as high in children as in adults, reaching 0.9% of this population [1].

CD is a chronic and systemic autoimmune enteropathy triggered by gluten (present in wheat, barley, and rye) ingestion in genetically predisposed individuals. Its clinical manifestations go beyond the gastrointestinal system, and more extraintestinal clinical manifestations have been identified in the diagnosis, such as iron deficiency anaemia, malnutrition, growth deficit, delayed puberty, tooth enamel defect, headache, chronic fatigue, dermatitis herpetiformis, neuropathy, and liver dysfunction [2,3,4,5].

The gluten-free diet (GFD) is currently the only effective treatment, and its main objective is to promote the recovery of the intestinal mucosa, a process that can take months or even years. In children, although the histological response is observed in up to two years, clinical improvement can be seen within two to four weeks. In addition, the GFD plays a fundamental role in preventing long-term complications associated with untreated CD [5,6].

However, as it excludes a large part of the carbohydrate sources widely consumed and present in the diet of many cultures around the world (such as pasta, bread, cakes, cookies, etc.), the GFD is considered a strict and restrictive diet, and is a great challenge for patients [7]. Consequently, adherence to the GFD is one of the main challenges faced by children and adolescents with CD, and it can impair food attitudes and behaviours [1]. In children, adherence to a GFD mainly depends on family support, as caregivers are responsible for dietary management, and it also varies according to factors such as age at diagnosis, how caregivers manage treatment, and how much they know about CD. During the transition to adolescence, new barriers related to social, emotional, and psychological issues may pose additional risk factors for GFD adherence, especially as adolescents seek more autonomy in food choices and social interaction outside the family environment [8,9,10,11].

Studies estimate that adherence to the GFD in this population can vary between 23% and 98%, with a median rate of 78% [12]. Even when maintaining good adherence to a GFD, patients with CD are subject to episodes of involuntary dietary transgression due to cross-contact (when gluten from other products naturally contaminates gluten-free foods). This poses a risk to both health and quality of life, as it requires constant monitoring of food labels and preparation, especially when they need to eat out. Such circumstances can lead to the development of maladaptive attitudes and behaviours related to food control, which may act as precursors or even indicators of disorders [13,14]. Consequently, the individual’s relationship with eating inevitably changes after diagnosis—perhaps the only immediate certainty after CD diagnosis [15].

Although taking certain precautions to avoid cross-contact is necessary, hypervigilance in managing adherence to the GFD can become an exacerbated concern. This concern is mainly related to the possibility of cross-contact or accidental ingestion of gluten and the symptoms and consequences of gluten ingestion. There is, therefore, an overestimation of this risk and persistent thoughts about the negative consequences that may occur. Food hypervigilance poses a risk to the individual’s emotional and social aspects, since it is associated with symptoms of anxiety and fatigue due to the high demand imposed by the GFD. It includes rigid, repetitive, and obsessive behaviours regarding the possibility of gluten presence in foods prepared by others, resulting in compulsive label checking, strict control of the food environment, avoiding eating outside the home, restriction of social activities involving food, and the feeling of being different from peers [13,16,17,18].

Studies suggest that food hypervigilance and other maladaptive eating behaviours are associated with a worse quality of life in children and adolescents with CD, especially concerning emotional and psychological aspects [11,14,16,19,20]. In light of this, Maddison-Roberts (2023) [13] developed a specific instrument to assess food attitudes and behaviours in children and adolescents with CD in the United Kingdom (UK): the Child Coeliac Disease Food Attitudes and Behaviours Scale (Child CD-FAB). In the pilot study to validate the Child CD-FAB, significant correlations were found between food hypervigilance and lower quality of life among children and adolescents with CD in the UK, highlighting the need for specific instruments to measure this impact and the importance of this knowledge in different countries to support the targeting of public policies and health services for this population.

In Brazil, however, there is no specific instrument to evaluate food attitudes and behaviours of children and adolescents with CD, which represents a relevant gap for research and clinical practice. Therefore, this study aims to translate the Child CD-FAB into Brazilian Portuguese and evaluate its reproducibility and internal consistency. By providing a validated instrument to assess food attitudes and behaviours in this population, we hope to enable future research and assist health professionals in approaching and supporting these patients.

## 2. Materials and Methods

### 2.1. Study Design and Ethical Approval

This study was conducted following the Declaration of Helsinki and approved by the Research Ethics Committees of the Faculty of Health Sciences of the University of Brasília CEP/FS (CAAE: 78781624.8.0000.0030). It also has permission from the author of the original instrument (Child CD-FAB) for translation and validation for use within the Brazilian population. The volunteers and their legal guardians received and signed a consent form. To ensure the validity of the translation and cultural adaptation of the Child CD-FAB, the methodological process followed three steps: (i) translation and cultural adaptation of the Child CD-FAB following previously established protocols; (ii) pre-test to assess item clarity and comprehension; and (iii) evaluation of the psychometric properties of the CD-FAB-BR.

### 2.2. The Child CD-FAB

Initially developed in British English, the Child CD-FAB is a unidimensional measure consisting of 14 items that aim to assess food attitudes and behaviours associated with adherence to the GFD in children and adolescents with CD. The response options are arranged on a 7-point Likert scale from 1 “strongly agree” to 7 “strongly disagree”. The total score of the Child CD-FAB is obtained by summing the responses to each item, reversing the values of the last two items. The final score ranges from 14 to 98, with higher scores indicating greater hypervigilance towards the GFD. The instrument demonstrated excellent internal consistency (Cronbach’s alpha = 0.93) in its original version, with all item-total correlations above 0.30, reinforcing the instrument’s robust psychometric structure.

In addition to demonstrating strong internal consistency, the original version also presented consolidated evidence of external validity. Higher scores on the instrument correlated positively with anxiety symptoms, stronger illness perception and control beliefs, and increased food neophobia. Furthermore, negative correlations were observed with quality of life indicators, which reinforces its psychometric foundation already established in the international literature.

### 2.3. Translation and Cultural Adaptation of the Child CD-FAB

**Figure 1:**
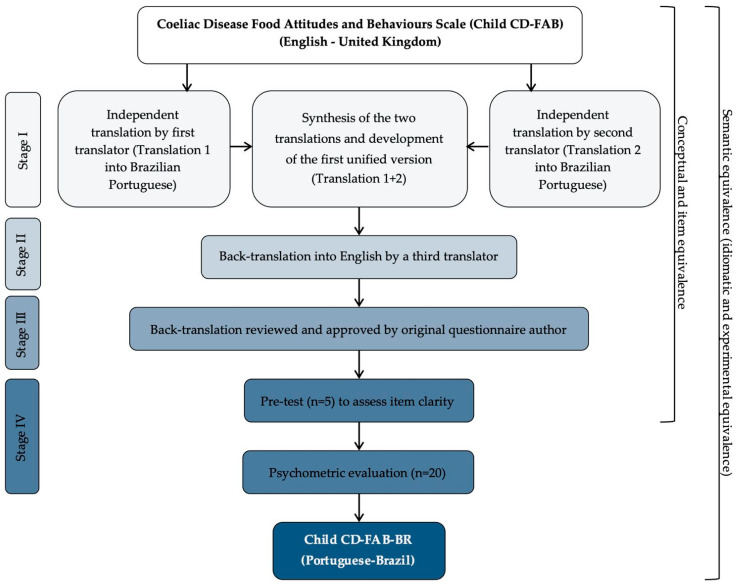
Sequential stages of the Child CD-FAB translation process from the original English version to the Brazilian Portuguese version.

The translation and adaptation stage followed the methodological guidelines proposed by Beaton et al. (2000) [21], comprising four sequential stages as illustrated in Figure 1: (i) translation and synthesis of translations; (ii) back-translation; (iii) sending the produced material to the developers of the original instrument; (iv) pre-testing and evaluation of the psychometric properties of the adapted instrument.

Initially, two independent Brazilian bilingual translators without prior knowledge of the tool translated the Child CD-FAB, including the instructions for correct completion and the items. One of the translators is from the theoretical field related to the instrument, while the other is not. Both followed the recommendation that the translation preserve the popular language and be easy to understand, considering the target audience. Then, in a collaborative effort, three researchers discussed the two translated versions to achieve a unified version. This unified version was sent to a foreign bilingual translator for the retranslation stage. The translator had no prior contact with the original instrument and did not participate in the previous stages. The back-translation version was subsequently analyzed and validated by the author of the original instrument.

### 2.4. Pre-Test

With the final version of the translated instrument, a pre-test was carried out to assess the clarity and comprehension of the translated items, using a convenience sample (*n* = 5) composed of patients treated at the Coeliac Disease Outpatient Clinic of the University Hospital of Brasília (HUB). The inclusion criteria were as follows: Brazilian children and adolescents aged between 8 and 16 years, diagnosed with CD for at least 12 months, residing in Brazil, and whose parents or caregivers agreed to their participation in the study.

Given the translation process of an instrument for children and adolescents, particularly those diagnosed with CD (which limits the sample in terms of age range and diagnosis), the pre-test protocol recommended for questionnaires translated for the same age group was adopted. In this protocol, the pre-test must be conducted with at least four native speakers who are unfamiliar with the tool. Then, each participant evaluates their understanding of the meaning of each item. Based on the participants’ responses, the translated version was evaluated and the items were adjusted. The final translated version of each item was recorded in the translation table, which was then shared and discussed with the author of the instrument before the final approval of the translation [22,23,24].

An invitation was sent to the child’s caregivers, followed by scheduling of a remote structured interview, conducted via WhatsApp video call, with the child and at least one caregiver. The interview initially included identification questions (name initials, age, age at diagnosis, and state of residence) to confirm inclusion criteria. Then, participants evaluated each item of the translated instrument for clarity, using a Likert scale of 1 to 5 points, where 1 corresponded to “I did not understand at all” and 5 corresponded to “I understood completely”. Items were presented individually and, whenever a score between 1 and 3 was assigned, participants were invited to express doubts or suggest adjustments. Items with a score of 4 or 5 by at least 80% of the participants were considered satisfactory in terms of clarity. Items with a score below this percentage were marked for revision.

### 2.5. Psychometric Evaluation of the Child CD-FAB-BR

Reproducibility and internal consistency were evaluated using responses obtained through a test–retest procedure. In the instrument evaluation process, before applying it to a large sample, it is crucial to test the reproducibility (reliability) in a small sample of participants [25]. Reproducibility evaluates the degree of correlation between any two measurements taken from the same subject [25]. This step is essential before performing the external validation of the instrument with a representative sample [25].

The test–retest was conducted with a convenience sample of 20 participants who met the inclusion criteria (the same inclusion criteria used in the pre-test step). Participants were recruited through invitations sent via messaging applications (WhatsApp), social networks (Instagram and Facebook), dissemination by the Coeliac Association among CD researchers in Brazil, and through patients attending outpatient clinics for diagnosis and treatment of CD. Those who met the inclusion criteria were asked to complete the instrument twice: once initially and again after an interval of 48 h to 15 days, without prior knowledge that they would be asked to respond a second time to assess reproducibility [25,26,27,28].

Responses were recorded using structured questionnaires, available on the Google Forms virtual platform, containing the following: (i) sociocultural and health information, including the initials of the child’s name, age, self-reported race/color, age at diagnosis of CD, medical confirmation of the diagnosis, and state of residence; (ii) adherence to the gluten-free diet, with the question “Do you follow a gluten-free diet?”, whose response options were always, sometimes, rarely and never; (iii) concern about gluten consumption, with the question “Do you feel worried about foods that contain gluten?”, with yes/no answers; and (iv) the Child CD-FAB-BR.

### 2.6. Statistical Analysis

Descriptive statistics are presented as frequencies and percentages (%) for categorical variables, and as means and standard deviations (SDs) for quantitative variables. The instrument’s reproducibility (test–retest reliability) was verified using the intraclass correlation coefficient (ICC). The ICC was evaluated using a two-way mixed-effects model, assessing absolute agreement and considering the mean of the observations. ICC values between 0.75 and 0.90 and values greater than 0.90 indicate good and excellent test–retest reliability, respectively [29]. The internal consistency of the instrument was verified using Cronbach’s alpha coefficient (α), with values of α greater than 0.7 indicating adequate internal consistency [30]. All statistical analyses were performed using IBM SPSS version 22.0 software (SPSS Inc., Chicago, IL, USA).

## 3. Results

### 3.1. Translation and Cultural Adaptation of the Child CD-FAB

The results of the Child CD-FAB translation stages demonstrate the attention given to adaptation the instrument to the Brazilian cultural context. As shown in Table 1, during the translation process, specific expressions were discussed directly with the author of the original instrument to ensure cultural adaptation without compromising the original meaning. This ensured that the translated version maintained the meaning of the sentences while being understandable to the target audience. Specifically, a type of expression appearing in items 7, 10 and 12 required discussion. Additionally, item 13 underwent a cultural adaptation through a different process, which is not reflected in Table 1 because it was not related to back-translation discrepancies but rather to cultural contextualization needs.

### 3.2. Pre-TestEvaluation

The pre-test (*n* = 5) was conducted to verify the clarity and comprehension of the sentences in the translated instrument (Table S1—Characterization of pre-test participants). Of the 14 items, 12 presented a 100% comprehension rate. The items related to “concern when sitting near other people who are eating gluten” and “difficulty eating foods that resemble those with gluten” presented an agreement rate of 80%, a value adequate to validate the items. The results indicated that the translated instrument is clear and comprehensible for the Brazilian target audience.

### 3.3. Reproducibility and Internal Consistency of the Child CD-FAB

Initially, 47 parents or caregivers of children expressed interest in participating in the study. Of these, 4 participants were excluded because they did not meet the inclusion criteria, 7 withdrew after receiving the link to participate, and 13 did not receive the link because they did not respond to the initial contact after expressing interest. Thus, 23 individuals fully completed the initial instrument (test) and were included in the first stage.

Of these 23 participants, 20 completed the instrument again when requested (retest) (Table S2—Characterization of test–retest participants; Table S3—Characterization of participants regarding diagnosis, adherence to a gluten-free diet and concern with food). Most participants were female (70%), with a mean age of 10.75 ± 2.36 years. The average interval between responses was 169.26 ± 85.85 h (approximately 7 days). Data obtained from the 20 participants who completed test and retest were subjected to reproducibility analysis using the intraclass correlation coefficient (ICC). The ICC obtained for Child-CD-FAB-BR was 0.923, indicating excellent agreement between the two applications. The mean total score obtained in the test was 47.5 (*SD* = 18.0), while in the retest, it was 51.5 ± 19.5, with a mean difference of only 4 points between applications.

In addition to temporal stability, the instrument’s internal consistency was also assessed using Cronbach’s alpha coefficient, calculated from the responses of the initial application (test). The value obtained (α = 0.892) indicates that the instrument items correlated highly, demonstrating that they cohesively assess the proposed construct. The final version of the Child CD-FAB-BR is available in the Supplementary Material (Table S4).

## 4. Discussion

This study is the first to translate, culturally adapt, and evaluate the psychometric properties of a specific instrument to assess the food attitudes and behaviours of children and adolescents with CD in Brazil. Although there are tools to assess disordered eating behaviours, recent studies have highlighted that these measures fail to adequately capture the specificities observed in individuals with CD [31]. Beliefs related to perceived risks around cross-contact and food safety contribute to restrictive and avoidant eating behaviours, marked by food hypervigilance. Even though they are based on the need to follow a GFD, these behaviours can negatively affect individuals’ quality of life and well-being [11,16,18]. Therefore, using a specific tool to assess the food attitudes and behaviours of people with CD, translated and culturally adapted for each country, enables the collection of concrete and specific data that will encourage the formulation of public policies for this population and enable comparison between different countries, thereby allowing for the identification of strengths and weaknesses in this field.

To understand the specificities of food attitudes and behaviours of individuals with CD, the Coeliac Disease Food Attitudes and Behaviours Scale (CD-FAB) was developed for the adult CD population [19]. Based on four dimensions related to CD and GFD—food handling, trust, attitude towards risk, and food safety—the tool was shown to be sensitive in identifying behaviours specific to CD, which are not covered in more generic measures. Recognizing the need to assess these same behaviours in younger age groups, the Child CD-FAB was developed as an adaptation of the CD-FAB, reformulating the items to make them appropriate for the vocabulary, experiences, and realities of children and adolescents [13].

Considering the lack of specific tools to assess the food attitudes and behaviours of children and adolescents with CD in Brazil, this study translated, culturally adapted, and validated the Child CD-FAB into Brazilian Portuguese. The translation and cultural adaptation steps followed the methodological protocol proposed by Beaton et al. (2000) [21], ensuring rigor and equivalence between the original and translated versions. The active participation of the author of the original version of the Child CD-FAB in this process was an important differential, as it enabled the discussion of culturally loaded terms, which was essential to achieve satisfactory semantic and conceptual equivalence.

Discussion regarding the original and re-translated versions facilitated the refinement of the Brazilian version while ensuring it retained the same meaning as the original instrument. Table 1 shows that three items (7, 10, and 12) presented differences between the original and the re-translated English versions, which were discussed among the authors. Consequently, the term “being glutened” was modified to achieve better comprehension in Brazil. The term “*contaminada com glúten*” was used instead of a literal translation of “being glutened”, which appears in items 7, 10, and 12. The modification was approved by the author of the original instrument because “*contaminada com glúten*” is the expression most commonly used to describe gluten cross-contact situations for the target audience, which was later confirmed in the pre-test when the participants naturally understood the sentence.

Beyond terminology adjustments, another modification involved the decision to adopt a cultural adaptation rather than a literal translation for item 13. The original version uses the term “meals” (*refeições* in Brazilian Portuguese) to emphasize full meals prepared on-site, as many British children perceive ready-made and packaged snacks as safer. In Brazilian Portuguese, however, the expression “*sair para comer*” was chosen—even without a literal equivalent to “meals”—as it naturally refers to eating meals in places such as restaurants and canteens, ensuring semantic equivalence with the intended meaning of the original item.

The pre-test results confirmed the semantic and cultural adequacy of the Child CD-FAB-BR, showing that participants satisfactorily understood the items. The language used in the instrument was clear, accessible, and compatible with the level of understanding of Brazilian children and adolescents with CD, aged between 8 and 16 years old [21]. The cultural adaptation process demonstrated that, despite the apparent clarity and objectivity of certain items, it is essential to consider cultural and linguistic aspects that may influence how the content is interpreted. These adjustments are recognized in the literature as a fundamental step in the cultural adaptation of instruments, especially in paediatric populations [32,33].

Reliability tests, which assess the ability of a tool to produce consistent results when applied under similar conditions, are a necessary step before large-scale application, and they can be conducted with a reduced sample [34]. In this study, reliability was verified through reproducibility and internal consistency, using test–retest. Reproducibility, in this context, represents the temporal stability of the responses and should be assessed with an interval of 2 to 14 days between applications [35]. The present study’s average interval was seven days, demonstrating compatibility with these guidelines and adequacy for evaluating response stability [30,35]. According to the literature, ICC values above 0.75 are considered satisfactory for tools with this purpose [30]. The value obtained in this study (ICC = 0.923) indicates excellent reliability of the Child CD-FAB-BR regarding the stability of responses over time.

However, since memory bias or small real changes in health status between applications can influence the results, an internal consistency analysis was also conducted. Calculated using Cronbach’s alpha, this measure expresses the degree of correlation between the items of an instrument, that is, the extent to which the items assess the same construct [30,36]. In this study, the value obtained was 0.892, indicating a strong correlation between the items of the Child CD-FAB-BR, since values above 0.7 are considered satisfactory for population research [30,36]. These results demonstrate that the instrument has adequate psychometric properties and that the combination of a high ICC with a consistent Cronbach’s alpha reinforces its reliability for assessing food attitudes and behaviours. Confirmation of these psychometric properties allows for use of this tool in clinical practice, which may strengthen nutritional monitoring and expand comprehensive care for children and adolescents with CD. The tool can assist health professionals in the early identification of disordered eating behaviours, including emotional distress, food restriction or avoidance, and food hypervigilance—aspects that generic tools may not assess. It will allow health professionals to tailor their approach to each patient, supporting the development of adaptive self-management strategies and contributing to the prevention and/or treatment of disordered eating behaviours.

### Limitations

Some potential limitations should be considered when interpreting the results and planning future research. The tool covers the age range of 8 to 16 years, excluding groups such as younger children and older adolescents, whose eating experiences may differ. In Brazil, children are considered individuals aged 0 to 12 years old, while adolescents are those aged 13 to 18 years old [37]. We opted to use the same age range as the original version to preserve equivalence and allow for comparisons between different populations. However, further studies could be conducted to verify instrument validity in 16–18 y/o individuals. Regarding younger children, systematic reviews have shown that children under eight years of age face cognitive and linguistic limitations when using self-administered instruments (SDIs) due to their limited long-term memory, attention, reading comprehension, and judgment skills. Therefore, it is suggested that reporting for younger children be performed by proxy or administered by an interviewer [38,39].

In this study, the presence of parents or caregivers during the instrument administration was essential to ensure comprehension of the items, particularly among younger children. However, such mediation may have influenced some responses, representing another methodological limitation common in studies using SDIs with paediatric populations. To reduce this potential bias, the Informed Consent Form (ICF) explicitly highlighted that responses should exclusively reflect the child’s or adolescent’s perceptions. Future studies could adopt strategies to systematically evaluate the influence of caregiver presence, such as parallel protocols with and without their direct participation.

Another limitation relates to the use of a convenience sample. While this approach allows for easy tracking of participants and ensures they receive an invitation for a second response promptly, it may have limited sample diversity and resulted in a small and homogeneous sample. Despite this, 20 duplicated answers have been demonstrated to be enough [28]. Additionally, the final sample was predominantly composed of female participants, a pattern similar to that identified in other studies involving individuals with CD [11,16,20,40,41].

Furthermore, this study focused on psychometric analyses of reproducibility and internal consistency, which required smaller samples but did not include analyses associated with other variables, tools, or external measures. Finally, the cross-sectional design of the study limits conclusions about changes in food attitudes and behaviours over time. Longitudinal studies would be particularly valuable to capture variations in psychological adjustments, dietary management, and adherence challenges throughout childhood and adolescence.

## 5. Conclusions

This study successfully translated, culturally adapted, and validated the Child CD-FAB into Brazilian Portuguese, resulting in the first tool designed to assess the food attitudes and behaviours of children and adolescents with CD in Brazil. The psychometric findings indicated excellent reliability of the Brazilian version. Therefore, it is expected that, with the availability of this tool, health professionals can now better identify and treat disordered eating patterns more effectively. In the long term, this advancement may contribute to the strengthening of public policies aimed at nutritional care for this population. To achieve these goals, it is necessary to expand the application of the Child CD-FAB-BR and integrate it with other variables and tools. Future studies should focus on large-scale application of the Child CD-FAB-BR to enable external validation, perform additional analyses, expand knowledge about the eating patterns of children and adolescents with CD, and deepen the understanding of the factors that shape food attitudes and behaviours in this population. A future nationwide study can contribute not only to strengthening the evidence for the scale’s validity but also to a deeper understanding of the eating attitudes and behaviors of children and adolescents with CD in the Brazilian context.

## Figures and Tables

**Table 1:** The original and translated instrument versions of the Child CD-FAB, including back-translation, comments, and final adjustments.

Item	Original	Translation	Back-Translation	Comments	Response
My coeliac disease makes me feel…/*A doença celíaca me faz sentir*…					
1	worried when I’m sat near other people who are eating gluten.	*preocupado(a) quando estou sentado(a) perto de outras pessoas que estão comendo glúten.*	worried when I am seating near other people who are eating gluten		
2	afraid to eat outside my home (e.g., school, clubs, restaurants, friend’s house).	*com medo de comer fora de casa (por exemplo na escola, em clubes, em restaurantes, na casa de amigos).*	scared of eating outside of home (for example in school, clubs, restaurants, friend’s houses).		
3	afraid to touch foods with gluten in them.	*com medo de tocar em alimentos que contenham glúten.*	scared of touching food that contains gluten.		
4	worried when eating with people I don’t know well (e.g., new friends).	*preocupado(a) ao comer com outras pessoas que não conheço bem (por exemplo novos amigos).*	worried when eating around people that I do not know well (for example, new friends).		
5	worried about going to events where there is food (e.g., parties, sleepovers)	*preocupado(a) em ir a eventos onde tem comida (por exemplo festas, festa do pijama).*	worried about going to events where there is food (for example, parties, pyjama parties).		
Because of coeliac disease…/*Por causa da doença celíaca*…					
6	I find it hard to eat gluten-free foods that look like foods with gluten in them (e.g., gluten-free pasta, gluten-free cakes).	*acho difícil comer alimentos sem glúten que pareçam com alimentos com glúten (por exemplo macarrão sem glúten, bolos sem glúten).*	I find it difficult to eat gluten-free food that looks like food containing gluten (for example, gluten-free pasta, gluten-free cakes).		
7	I worry about all the different ways that my food could be glutened.	*eu me preocupo com todas as formas pelas quais minha comida pode ser contaminada com glúten.*	I worry about all the ways in which my food can get contaminated with gluten.	We found that most children called being contaminated with gluten “being glutened”, so used this term to stay close to their language and make it easier for them to understand. I’d be interested in what children call this in Brazil, and perhaps using this term?	There is no specific term equivalent to “being glutened” in Brazil. Children and adolescents usually use the expression “contaminated with gluten” to describe the situation. We kept this expression to ensure understanding within the Brazilian cultural context.
8	I only eat food that my parents or carer have prepared.	*eu só como alimentos que meus pais ou cuidadores tenham preparado.*	I only eat food that my parents or carers have cooked.		
9	I find it hard to trust gluten-free food prepared by other people (e.g., friend’s parents, restaurants).	*eu acho difícil confiar em alimentos sem glúten preparados por outras pessoas (por exemplo pais de amigos, restaurantes).*	I find it hard to trust gluten-free food cooked by other people (for example, friend’s parents, restaurants).		
10	Being glutened in the past has stopped me from enjoying eating out.	*ter ingerido glúten sem saber no passado me impediu de gostar de comer fora.*	Having ingested gluten without knowing in the past has stopped me from enjoying eating out.	As above, I wonder whether children are familiar with the terminology “ingested gluten” or whether there is another term they are more familiar with that might make it easier for them to understand? Nonetheless, this has the same meaning.	There is no specific term equivalent to “being glutened” in Brazil. Children and adolescents usually use the expression “contaminated with gluten” to describe the situation. We kept this expression to ensure understanding within the Brazilian cultural context.
11	I avoid eating food prepared by other people (e.g., friend’s parents).	*eu evito comer alimentos preparados por outras pessoas (por exemplo pais de amigos).*	I avoid eating food that is cooked by other people (for example, friend’s parents)		
12	I try to protect my food while eating to stop it from being glutened.	*eu tento proteger minha comida enquanto como para evitar que ela seja contaminada com glúten.*	I try to protect my food while I eat it to avoid it being contaminated with gluten.	As above, regarding whether children understand and are familiar with the word “contaminated”. Nonetheless, this has the same meaning.	There is no specific term equivalent to “being glutened” in Brazil. Children and adolescents usually use the expression “contaminated with gluten” to describe the situation. We kept this expression to ensure understanding within the Brazilian cultural context.
Even though I have coeliac disease…/*Mesmo tendo a doença celíaca*…					
13	I enjoy going out for meals.	*eu gosto de sair para comer.*	I like going out for a meal		
14	I am comfortable eating gluten-free food prepared by other people (e.g., a friend or family member).	*eu me sinto confortável para comer alimentos sem glúten preparados por outras pessoas (por exemplo amigos ou familiares).*	I feel comfortable to eat gluten-free food cooked by other people (for example, friends or family).		

## Data Availability

The original contributions presented in this study are included in the article. Further inquiries can be directed to the corresponding author.

## Supplementary Materials

The following supporting information can be downloaded at https://www.mdpi.com/article/10.3390/nu17162704/s1. Table S1: Characterization of pre-test participants; Table S2: Characterization of test–retest participants; Table S3: Characterization of participants regarding diagnosis, adherence to a gluten-free diet and concern with food; and Table S4: Final version of the Child CD-FAB-BR.

## Abbreviations

The following abbreviations are used in this manuscript:

CAAE	Certificate of Presentation for Ethical Assessment
CD	Coeliac Disease
CD-FAB	Coeliac Disease Food Attitudes and Behaviours Scale
GFD	Gluten-Free Diet
HUB	Hospital of Brasília
ICF	Informed Consent Form
ICC	Intraclass Correlation Coefficient
SDI	Self-Administered Instruments
UK	United Kingdom

## References

[1] Rubio-Tapia A., Hill I.D., Semrad C., Kelly C.P., Lebwohl B. (2023). American College of Gastroenterology Guidelines Update: Diagnosis and Management of Celiac Disease. Am. J. Gastroenterol..

[2] Ludvigsson J.F., Leffler D.A., Bai J.C., Biagi F., Fasano A., Green P.H.R., Hadjivassiliou M., Kaukinen K., Kelly C.P., Leonard J.N. (2013). The Oslo Definitions for Coeliac Disease and Related Terms. Gut.

[3] Lebwohl B., Sanders D.S., Green P.H.R. (2018). Coeliac Disease. Lancet.

[4] Lebwohl B., Rubio-Tapia A. (2021). Epidemiology, Presentation, and Diagnosis of Celiac Disease. Gastroenterology.

[5] Sahin Y. (2021). Celiac Disease in Children: A Review of the Literature. World J. Clin. Pediatr..

[6] Kurppa K., Mulder C.J., Stordal K., Kaukinen K. (2024). Celiac Disease Affects 1% of Global Population: Who Will Manage All These Patients?. Gastroenterology.

[7] de Oliveira D.C.L., da Silva V.M.B., da Silva L.M.C. (2022). Desafios Da Adesão à Dieta Sem Glúten. Res. Soc. Dev..

[8] Simón E., Molero-Luis M., Fueyo-Díaz R., Costas-Batlle C., Crespo-Escobar P., Montoro-Huguet M.A. (2023). The Gluten-Free Diet for Celiac Disease: Critical Insights to Better Understand Clinical Outcomes. Nutrients.

[9] See J.A., Kaukinen K., Makharia G.K., Gibson P.R., Murray J.A. (2015). Practical Insights into Gluten-Free Diets. Nat. Rev. Gastroenterol. Hepatol..

[10] Ludvigsson J.F., Agreus L., Ciacci C., Crowe S.E., Geller M.G., Green P.H.R., Hill I., Hungin A.P., Koletzko S., Koltai T. (2016). Transition from Childhood to Adulthood in Coeliac Disease: The Prague Consensus Report. Gut.

[11] Cadenhead J.W., Wolf R.L., Lebwohl B., Lee A.R., Zybert P., Reilly N.R., Schebendach J., Satherley R., Green P.H.R. (2019). Diminished Quality of Life among Adolescents with Coeliac Disease Using Maladaptive Eating Behaviours to Manage a Gluten-Free Diet: A Cross-Sectional, Mixed-Methods Study. J. Hum. Nutr. Diet..

[12] Myléus A., Reilly N.R., Green P.H.R. (2020). Rate, Risk Factors, and Outcomes of Nonadherence in Pediatric Patients With Celiac Disease: A Systematic Review. Clin. Gastroenterol. Hepatol..

[13] Maddison-Roberts H. (2023). A Thesis of Clinical Research and Practice: Part A: Understanding the Psychological Experiences of Children and Young People with Coeliac Disease and Their Relationship with Food. Ph.D. Thesis.

[14] Gholmie Y., Lee A.R., Satherley R.M., Schebendach J., Zybert P., Green P.H.R., Lebwohl B., Wolf R. (2023). Maladaptive Food Attitudes and Behaviors in Individuals with Celiac Disease and Their Association with Quality of Life. Dig. Dis. Sci..

[15] Falcomer A.L., Luchine B.A., Gadelha H.R., Szelmenczi J.R., Nakano E.Y., Farage P., Zandonadi R.P. (2020). Worldwide Public Policies for Celiac Disease: Are Patients Well Assisted?. Int. J. Public Health.

[16] Wolf R.L., Lebwohl B., Lee A.R., Zybert P., Reilly N.R., Cadenhead J., Amengual C., Green P.H.R. (2018). Hypervigilance to a Gluten-Free Diet and Decreased Quality of Life in Teenagers and Adults with Celiac Disease. Dig. Dis. Sci..

[17] Macedo L., Catarino M., Festas C., Alves P. (2024). Vulnerability in Children with Celiac Disease: Findings from a Scoping Review. Children.

[18] Maddison-Roberts H., Jones C., Satherley R.M. (2025). Gluten-Free Diet Management and Well-Being in Children with Celiac Disease: A Qualitative Study. Pediatr. Allergy Immunol..

[19] Satherley R.M., Howard R., Higgs S. (2018). Development and Validation of the Coeliac Disease Food Attitudes and Behaviours Scale. Gastroenterol. Res. Pract..

[20] Karwautz A., Wagner G., Berger G., Sinnreich U., Grylli V., Huber W.D. (2008). Eating Pathology in Adolescents with Celiac Disease. Psychosomatics.

[21] Beaton D.E., Bombardier C., Guillemin F., Ferraz M.B. (2000). Guidelines for the Process of Cross-Cultural Adaptation of Self-Report Measures. Spine.

[22] Dusi R., Botelho R.B.A., Nakano E.Y., de Queiroz F.L.N., Zandonadi R.P. (2023). Translation of the Satter’s Division of Responsibility in Feeding Questionnaire into Brazilian Portuguese: A Cross-Sectional Study. Nutrients.

[23] Lohse B., Satter E. (2021). Use of an Observational Comparative Strategy Demonstrated Construct Validity of a Measure to Assess Adherence to the Satter Division of Responsibility in Feeding. J. Acad. Nutr. Diet.

[24] Lohse B., Mitchell D.C. (2021). Valid and Reliable Measure of Adherence to Satter Division of Responsibility in Feeding. J. Nutr. Educ. Behav..

[25] Bartlett J.W., Frost C. (2008). Reliability, Repeatability and Reproducibility: Analysis of Measurement Errors in Continuous Variables. Ultrasound Obstet. Gynecol..

[26] Streiner D.L. (2003). Starting at the Beginning: An Introduction to Coefficient Alpha and Internal Consistency. J. Pers. Assess..

[27] Streiner D.L. (2003). Being Inconsistent About Consistency: When Coefficient Alpha Does and Doesn’t Matter. J. Pers. Assess..

[28] Bujang M.A., Omar E.D., Foo D.H.P., Hon Y.K. (2024). Sample Size Determination for Conducting a Pilot Study to Assess Reliability of a Questionnaire. Restor. Dent. Endod..

[29] Koo T.K., Li M.Y. (2016). A Guideline of Selecting and Reporting Intraclass Correlation Coefficients for Reliability Research. J. Chiropr. Med..

[30] Streiner D.L., Norman G.R. (2008). Health Measurement Scales: A Practical Guide to Their Development and Use.

[31] Wei Y., Wang Y., Yuan Y., Chen J. (2025). Celiac Disease, Gluten-Free Diet, and Eating Disorders: From Bench to Bedside. Foods.

[32] Stevelink S.A.M., Van Brakel W.H. (2013). The Cross-Cultural Equivalence of Participation Instruments: A Systematic Review. Disabil. Rehabil..

[33] Arestad K.E., MacPhee D., Lim C.Y., Khetani M.A. (2017). Cultural Adaptation of a Pediatric Functional Assessment for Rehabilitation Outcomes Research. BMC Health Serv. Res..

[34] Monti C.B., Ambrogi F., Sardanelli F. (2024). Sample Size Calculation for Data Reliability and Diagnostic Performance: A Go-to Review. Eur. Radiol. Exp..

[35] Marx R.G., Menezes A., Horovitz L., Jones E.C., Warren R.F. (2003). A Comparison of Two Time Intervals for Test-Retest Reliability of Health Status Instruments. J. Clin. Epidemiol..

[36] McDowell I. (2006). Measuring Health: A Guide to Rating Scales and Questionnaires.

[37] LTr Editora (1990). Estatuto Da Criança e Do Adolescente (Lei n. 8.069, de 13 de Julho de 1990).

[38] Lei R., Xiong J., Wang H., Li Y., Estill J., Li Q., Chen Y. (2024). Patient-reported Outcome Measures in Pediatrics: An Overview of Reviews. Pediatr. Discov..

[39] Coombes L., Bristowe K., Ellis-Smith C., Aworinde J., Fraser L.K., Downing J., Bluebond-Langner M., Chambers L., Murtagh F.E.M., Harding R. (2021). Enhancing Validity, Reliability and Participation in Self-Reported Health Outcome Measurement for Children and Young People: A Systematic Review of Recall Period, Response Scale Format, and Administration Modality. Qual. Life Res..

[40] Stroud C., Almilaji O., Nicholas D., Kirkham S., Surgenor S.L., Williams I., Snook J. (2020). Evolving Patterns in the Presentation of Coeliac Disease over the Last 25 Years. Frontline Gastroenterol..

[41] Shah S., Leffler D. (2010). Celiac Disease: An Underappreciated Issue in Womens Health. Women’s Health.

