# Supplementary material for: Adaptation of the Child Coeliac Disease Food Attitudes and Behaviours Scale (Child CD-FAB) into Brazilian Portuguese: Translation and Evaluation of Reproducibility and Internal Consistency
Source: Nutrients. 2025 Aug 21;17(16):2704. doi: 10.3390/nu17162704 (PMC12389570; doi:10.3390/nu17162704)
Supplement: Supplementary file 1 [file nutrients-17-02704-s001.zip › nutrients-3801825-supplementary.pdf]

Table S1. Characterization of the pre-test participants

Participants	Biological sex	Age (years)	Age at diagnosis (years)	State of residence
1	Female	10	3	Distrito Federal
2	Female	11	8	Distrito Federal
3	Female	14	1	Distrito Federal
4	Male	11	10	Distrito Federal
5	Female	12	10	Distrito Federal

 https://doi.org/10.3390/xxxxx

Table S2. Characterization of the test-retest participants

Participants	Biological sex	Age (years)	Age at diagnosis (years)	State of residence
1	Female	9	4	Distrito Federal
2	Male	14	2	Distrito Federal
3	Female	13	11	Distrito Federal
4	Female	11	8	Distrito Federal
5	Female	10	8	Distrito Federal
6	Female	9	7	Goiás
7	Female	9	4	Distrito Federal
8	Male	10	9	Rio Grande do Sul
9	Male	9	7	Minas Gerais
10	Female	10	8	São Paulo
11	Female	15	5	Distrito Federal
12	Male	8	6	Minas Gerais
13	Female	10	9	São Paulo
14	Female	9	2	Ceará
15	Male	9	8	Distrito Federal
16	Female	15	13	Minas Gerais
17	Female	12	5	Distrito Federal
18	Female	9	6	São Paulo
19	Female	9	8	Distrito Federal
20	Male	15	14	Pará

Table S3. Characterization of participants regarding diagnosis, adherence to a gluten-free diet and concern with food

		Sample (n=20)	
		**Frequency**	**%**
Diagnosis confirmed by a physician	Yes	20	100
	No	0	0
Adherence to the gluten-free diet	Always	17	85
	Sometimes	3	15
	Rarely	0	0
	Never	0	0
Concern about foods that may contain gluten	Yes	18	90
	No	2	10

Table S4. Final version of the Child CD-FAB-BR

Questionário de Atitudes e Comportamentos Alimentares de Crianças e Adolescentes com Doença Celíaca

Esse questionário explora como é ter a doença celíaca e seguir a dieta isenta de glúten.

Embora algumas pessoas também possam ter alergias ou intolerâncias alimentares, este questionário está perguntando sobre a doença celíaca.

Por favor, marque a Caixa que mostra o quanto você concorda com cada frase.

Não há respostas certas ou erradas.

	Concordo fortemente (1) 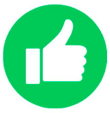	*Concordo (2)*	*Concordo um pouco (3)*	*Não concordo nem discordo (4)* 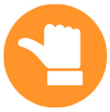	*Discordo um pouco (5)*	*Discordo (6)*	*Discordo totalmente (7)* 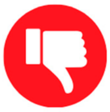
A doença celíaca me faz sentir…							
Preocupado(a) quando estou sentado(a) perto de outras pessoas que estão comendo glúten.							
Com medo de comer fora de casa (por exemplo na escola, em clubes, em restaurantes, na casa de amigos).							
Com medo de tocar em alimentos que contenham glúten.							
Preocupado(a) ao comer com outras pessoas que não conheço bem (por exemplo novos amigos).							
Preocupado(a) em ir a eventos onde tem comida (por exemplo festas, festa do pijama).							
Por causa da doença celíaca...							
Acho difícil comer alimentos sem glúten que pareçam com alimentos com glúten (por exemplo macarrão							

sem glúten, bolos sem glúten).							
Eu me preocupo com todas as formas pelas quais minha comida pode ser contaminada com glúten.							
Eu só como alimentos que meus pais ou cuidadores tenham preparado.							
Eu acho difícil confiar em alimentos sem glúten preparados por outras pessoas (por exemplo pais de amigos, restaurantes)							
Ter ingerido glúten sem saber no passado me impediu de gostar de comer fora.							
Eu evito comer alimentos preparados por outras pessoas (por exemplo pais de amigos).							
Eu tento proteger minha comida enquanto como para evitar que ela seja contaminada com glúten.							
Mesmo tendo a doença celíaca…							
*Eu gosto de sair para comer.**							
*Eu me sinto confortável para comer alimentos sem glúten preparados por outras pessoas (por exemplo amigos ou familiares).**							

*O escore total do CD-FAB Criança é obtido pela soma das respostas de cada item, invertendo os valores dos itens com *.*
